# Alectinib (Alecensa)-induced reversible grade IV nephrotoxicity: a case report and review of the literature

**DOI:** 10.1186/s13256-018-1849-y

**Published:** 2018-10-19

**Authors:** P Ramachandran, R Morcus, M Tahir, I Onukogu, B Spinowitz, Jen C Wang

**Affiliations:** 1 Division of Hematology/Oncology, Brookdale University Hospital Medical Center, Brooklyn, NY 11212 USA; 2 Division of Nephrology, New York Presbyterian Queens, Flushing, NY USA

**Keywords:** Acute renal failure, Alectinib, Adenocarcinoma, *ALK* rearrangement, Acute tubular necrosis, Non-small cell lung cancer, Case report

## Abstract

**Background:**

Lung cancer is among the top causes of cancer-related mortality in men and is the second most common cancer after breast cancer in women. There are approximately 234,030 new cases of lung cancer and 154,050 deaths from lung cancer in 2018 as per the latest American Cancer Society’s report. Alectinib, a more potent orally active tyrosine kinase inhibitor which was approved by the US Food & Drug Administration for anaplastic lymphoma kinase-positive lung adenocarcinoma, has been shown to have a reasonable safety profile when compared with other anaplastic lymphoma kinase-targeted therapy. As per research studies, grade 1 or 2 renal impairment has been reported but grade 4 renal toxicity due to alectinib has not been reported so far. We report a case of acute renal failure caused by alectinib which necessitated emergency dialysis. This is the first case report describing the severe renal toxicity of alectinib.

**Case presentation:**

We describe a case of 72-year-old Taiwanese man diagnosed with stage IV anaplastic lymphoma kinase-positive adenocarcinoma of the lung initially treated with crizotinib for over a year, which was switched to alectinib due to disease progression with brain metastasis. Within 6 weeks of starting alectinib, he developed acute renal failure needing emergency dialysis support. His renal failure was secondary to acute tubular necrosis and had a complete reversal within 7–10 days on withdrawing the medication. When he was re-challenged with alectinib, his creatinine started to worsen again which confirmed the renal toxicity of alectinib.

**Conclusions:**

This case emphasizes the uncommon adverse effect of the anaplastic lymphoma kinase-targeted therapy alectinib causing acute renal failure manifesting as acute tubular necrosis. Recognition of alectinib nephropathy requires a thorough drug history and knowledge of risk factors that lessen its margin of safety at therapeutic ingestions. Frequent monitoring of renal functions and early nephrology referral significantly reduce the mortality and morbidity of these patients.

## Background

Alectinib-induced acute renal failure (ARF) is a very entity and has not been reported so far in the literature. The two major research trials carried out to assess the safety of alectinib mostly reported only grade 1 and 2 renal impairment. ARF needing emergency dialysis support has not been reported so far.

Non-small cell lung cancer (NSCLC) accounts for approximately 85% of all lung cancers. Histological classification of NSCLC includes adenocarcinoma, squamous cell carcinoma (SCC), and large cell carcinoma. The most common subtype of NSCLC is adenocarcinoma which has targetable genomic changes. The tyrosine kinase receptors involved in the translocations of adenocarcinoma include anaplastic lymphoma kinase (*ALK*) (2p23), *ROS1* (6q22), and *RET* (10q11). These genomic translocations are most commonly seen in adenocarcinomas but can also be sometimes seen with squamous or adenosquamous variants. *EML4/ALK* is the most common ALK fusion found in lung adenocarcinoma (4 to 7% of cases) while *KIF5B/ALK* is the second common in frequency (0.5%) [1]. *ROS1* fusions and *RET* fusion genomics can exist in a small percentage of patients with NSCLC. Alectinib is a targeted therapy used in treating ALK-positive adenocarcinoma. It is a more potent second-generation inhibitor of ALK which was given an accelerated approval by the United States Food & Drug Administration (US FDA) in December 2015 for those who progressed on or are intolerant to crizotinib [2–4]. Alectinib was found to have more efficacy than crizotinib in the first-line setting in ALK-positive lung carcinoma. The most common adverse drug reactions with alectinib are weight gain (9.9%), photosensitivity reaction (5.3%), stomatitis (3.3%), interstitial lung disease (1.3%), and drug-induced liver injury (1.3%). Severe renal impairment is a very rare adverse effect and has been so far reported in < 1% of the cases. This case is unusual because it is the first case to report the reversible nephrotoxicity of Alecensa (alectinib) in a patient needing hemodialysis support. This is a very important clinical concern in metastatic lung cancer as it increases the morbidity and mortality of these patients and monitoring for these side effects is needed to achieve good outcomes.

## Case presentation

This is a case of a 72-year-old Taiwanese man who was diagnosed as having metastatic adenocarcinoma of the lung complicated by malignant right pleural effusion 2 years ago. He initially presented with weight loss, worsening cough, and worsening exertional shortness of breath for 3 months prior to presentation. He had a past medical history of type 2 diabetes mellitus, which was well controlled on insulin. He was an ex-smoker of tobacco with a tobacco smoking history of one pack a day for 10 years but he quit smoking tobacco 20 years ago. He also had a family history of non-Hodgkin lymphoma in his brother and breast cancer in his niece. He is retired and lives with his wife.

On examination, he was afebrile with heart rate of 70 beats/minute and with blood pressure (BP) of 130/80 mmHg. He appeared moderately built and was not in any respiratory distress. His respiratory examination was significant for dullness over the right middle and lower chest on percussion and was associated with reduced breath sounds on auscultation. His cardiovascular, abdominal, and neurological examinations were non-contributory.

His initial computed tomography (CT) scans demonstrated a middle lobe mass in his right lung and right lung pleural effusion. A positron emission tomography (PET) scan showed an increased uptake in the middle lobe mass in his right lung, subcarinal lymph nodes, several bilateral subcentimeter pulmonary nodules, and diffuse osseous metastasis. There was no evidence of brain metastasis as evidenced by magnetic resonance imaging (MRI) of his brain. He then had thoracentesis and pleural biopsy with the placement of a pleural catheter. The pleural biopsy was consistent with adenocarcinoma with an acinar pattern. Immunohistochemistry of the tumor cells was positive for cytokeratin (CK) 7, thyroid transcription factor 1 (TTF-1), and negative for CK20. A fluorescence *in situ* hybridization (FISH) showed evidence of ALK mutation (33% of cells positive for rearrangement). His final diagnosis was stage IV ALK + adenocarcinoma of the lung with metastasis to pleura, mediastinum, and bones. Before the information of ALK positivity was obtained, he was started on combination chemotherapy consisting of carboplatin, Alimta (pemetrexed), and Avastin (bevacizumab) of which he successfully completed five cycles. He was followed up regularly in the clinic every 4 weeks. He tolerated the chemotherapy and had a good response with 30% reduction in the lung mass size. He was later started on crizotinib 250 mg twice per day and had a significant response with improved tumor burden in his metastatic sites. He followed up in the clinic every 4 weeks initially for 6 months and then every 8 weeks for 1 year. During his follow-up visits, he remained stable with no evidence of disease progression. He remained on crizotinib for over a year and tolerated it well. During one of the follow-up clinic visits at around 18 months after diagnosis, an MRI scan of his brain was arranged due to a new symptom of headache; it showed numerous brain metastases which was consistent with progression of his disease. A decision was made to stop crizotinib and to start alectinib 600 mg twice daily coupled with cranial radiation.

**Fig. 1:**
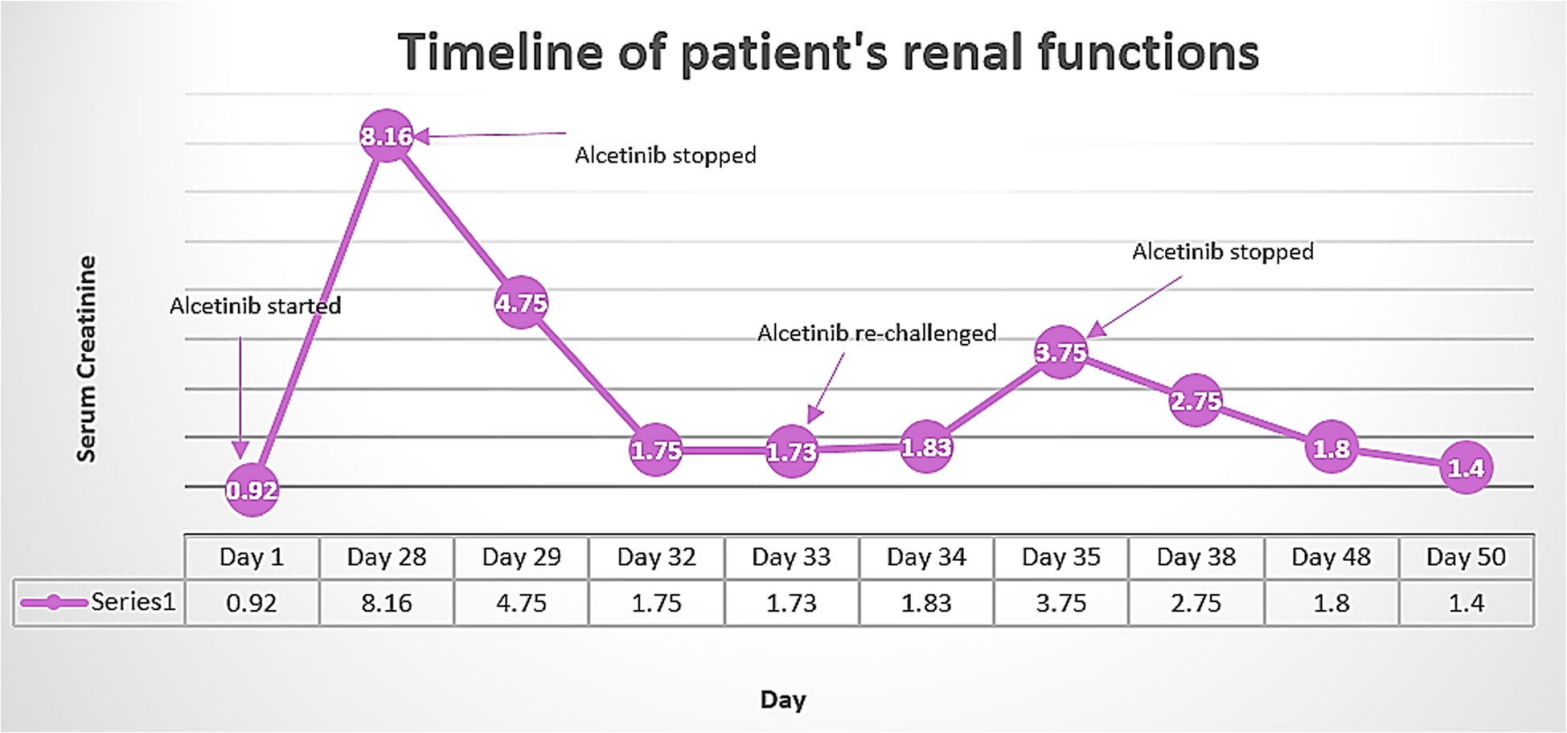
Timeline of patient’s renal functions

Within 5 weeks of starting alectinib, he developed ARF with his creatinine (Cr) increasing up to 8.16 mg/dL and blood urea nitrogen (BUN) to 113 mg/dl. He was anuric at presentation and his laboratory tests were consistent with hyperkalemia and acidosis with a potassium level of 7.1 mEq/L and bicarbonate (HCO_3_) of < 9 mmol/L. His renal workup revealed BUN/Cr ratio of 13, fractional excretion of sodium (FENa) of 16%, urine sodium of > 83 mEq/L, and urine osmolality of 334 mOsm/kg. His renal ultrasound did not show any evidence for obstruction (hydronephrosis). Table 1 illustrates the laboratory values and Fig. 1 illustrates the timeline of our patient’s renal functions.

**Table 1:** Laboratory values during alectinib therapy

	Baseline	After starting alectinib	1 week upon stopping alectinib
Creatinine (mg/dl)	1.0	8.16	1.75
BUN (mg/dl)	15	113	23
Potassium (mEq/L)	4.1	7.1	4.3
HCO_3_ (mmol/L)	24	8	24
eGFR	71	9	50

*BUN* blood urea nitrogen, *eGFR* estimated glomerular filtration rate, *HCO*_*3*_ bicarbonate

Since he was refractory to medical treatment, he required continuous venovenous hemodialysis (CVVH) due to the electrolyte imbalance. Alectinib was held on admission and dialysis was continued for 2 days until the electrolyte imbalance was corrected. Renal biopsy could not be performed as our patient refused.

His renal functions slowly recovered and Cr improved to 1.75 mg/dL within 2 days. He was re-challenged with alectinib at the same dose 24 hours after renal recovery. However, the medication had to be stopped again as his Cr started to worsen and rose to 3.6 mg/dL within 2 days of restarting alectinib. Currently, he is being treated with ceritinib, and his renal status has been stable with Cr levels ranging between 2 and 3 mg/dL. He also has no progression of his metastatic disease as evidenced by his recent imaging.

## Discussion

This case illustrates the sequence of renal impairment in a patient who was newly started on alectinib. It describes the acute rise in Cr within a few days of starting the medication which needed hemodialysis support to reverse the renal dysfunction. This case emphasizes the need for frequent monitoring of renal functions and avoiding other nephrotoxic drugs when using ALK inhibitors concomitantly.

Alectinib is an orally administered tyrosine kinase inhibitor which specifically targets *ALK* and *RET* gene rearrangements [5]^.^ The initial approval in 2015 by the US FDA was for treating patients with ALK cancers for both locally advanced and metastatic NSCLCs who had disease progression on crizotinib [3, 4]. The response rate has been very good when used as a first-line therapy accounting for approximately 93.5%. The median progression-free survival was shown to be 25.7 months when compared to 10.4 months with crizotinib. The incidence of brain metastasis was also shown to be lower when compared to crizotinib (9% versus 41%) [6]. Among the other therapies used in the treatment of ALK-positive NSCLC, alectinib has a reasonable safety profile, although the lack of controlled safety data limits this assessment. The common adverse reactions reported with alectinib include fatigue (41%), myalgia (29%) constipation (34%), and edema (30%). Serious side effects include interstitial lung disease, bradycardia, hepatotoxicity, and creatine phosphokinase (CPK) elevation.

The safety of alectinib was evaluated in 253 patients with ALK-positive NSCLC in two clinical trials: NP28761 and NP28673 [7]. The dose of 600 mg twice a day was used, and the median duration of exposure was 9.3 months. According to these trials, the genitourinary adverse effects accounted for < 6% of all side effects and included hematuria, nocturia, dysuria, proteinuria, urinary retention, urinary incontinence, azotemia, and an increase in Cr. Most of these events were grade 1 and 2 in severity, with grade ≥ 3 event (increased Cr) reported in only one patient (0.4%), where alectinib was discontinued [8].^.^ Studies about alectinib-induced renal toxicity are very limited. Dose reduction and treatment breaks overcome the adverse renal effects of ALK inhibitors providing clinical benefits for patients.

To the best of our knowledge, our current case report is the first one to describe the ARF and the clinical course of renal impairment encountered with alectinib. Although the exact mechanism of ARF with alectinib is not clear so far, there are theories which support a pre-renal or renal cause. From our experience, we postulate that the mechanism of ARF in our case could be secondary to acute tubular necrosis (ATN). The clinical euvolemia status with the absence of any underlying heart failure and lack of exposure to any nephrotoxic agents supported the diagnosis of ATN. The laboratory evidence of ATN included BUN/Cr ratio of 13, high FENa of 16% on presentation, urine sodium > 83 mEq/L, urinary osmolality of < 350 mOsm/kg, and no evidence of obstruction (hydronephrosis) on renal imaging.

While renal biopsy would be helpful in distinguishing among various renal pathologic entities (that is, glomerular versus tubulointerstitial disease versus thrombotic microangiopathy), it would not prove definitive cause and effect. The temporal relationship between the initiation of therapy with this drug and the development of renal dysfunction, as well as the absence of other causes of acute kidney injury (AKI), is supportive of the association of alectinib with the episode of renal failure.

It is interesting to note that alectinib which is excreted mostly by hepatobiliary route (98%) can still cause nephrotoxicity, despite the hepatobiliary route of metabolism. Due to the high renal blood flow to the kidney, exposure of tubular cells to the drug can be significant. Also, despite the low filtration of the given drug, luminal concentrations of the medication can be quite high due to fluid reabsorption throughout the tubule.

Nephrotoxicity associated with alectinib can be reduced by discontinuing the medication and re-challenging with a reduced dose to avoid dose-related nephrotoxicity [9]. The other measures which could reduce this complication would be to avoid volume depletion and dehydration in addition to avoiding the concomitant use of other nephrotoxic medications. Investigating further for other causes of renal failure would narrow down the differentials. Frequent monitoring of renal functions by trending Cr levels before initiating therapy and periodically every week would help to identify the patients at high risk of renal dysfunction. These patients could be followed up monthly if their Cr levels remained stable. Early nephrology referral is considered helpful to reduce the mortality and the hospitalizations in these patients.

## Conclusions

Our patient’s case demonstrates an extremely rare side effect of alectinib. Alectinib-induced nephrotoxicity has so far been reported in < 4% of patients in research studies [9]. Median time to cause grade III–IV renal impairment was around 4 months. In contrast, our patient developed grade IV renal impairment (Cr > 6, upper limit of normal) within 6 weeks of starting the treatment and required emergency dialysis. ATN seems to be the most common underlying mechanism of alectinib-induced nephrotoxicity. It was found to be completely reversible when the inciting medication was discontinued. Although renal toxicity is not a common side effect, this case emphasizes consideration of alectinib-induced nephrotoxicity as an underlying reason for azotemia during alectinib therapy.
